# “You have to love the party setting”: an ethnography of the blurred lines between roles and experiential knowledge in a French harm reduction collective working in the party setting

**DOI:** 10.1186/s12954-025-01209-9

**Published:** 2025-05-07

**Authors:** Serena Garbolino, Marie Dos Santos, Ombline Pimond, Lionel Sayag, Nicolas Khatmi, Perrine Roux

**Affiliations:** 1 Aix Marseille Univ, Inserm, IRD, SESSTIM, Sciences Economiques & Sociales de la Santé & Traitement de l’Information Médicale, ISSPAM,, Marseille, France; 2 Assocation P.R.O.S.E.S, 63 avenue de la Résistance, Montreuil, 93100 France

**Keywords:** Party setting, Harm reduction, Experiential knowledge, Role blurring, Ethnography

## Abstract

**Background:**

The party setting is a dynamic social environment where the world of drug use, the role of music, and a multiplicity of social interactions all converge, often marked by the disruption of social and temporal norms and rules. People who use drugs (PWUD) in the party setting are rarely targeted by institutional harm reduction (HR) interventions despite the many risks specific to this setting. *InterCAARUD Festif Île-de-France* (IFI) is a collective of French HR associations implementing interventions in the party setting for over a decade through coordinated teams of HR volunteers. We investigated the organization of the IFI collective with a view to acquiring a better understanding of the specific features that enable it to provide relevant HR interventions in the party setting.

**Methods:**

We collected data over nine months using ethnographic methods (participant observations, photography, field notes and informal interviews), focus groups and semi-structured interviews. We analyzed these data using a thematic analysis.

**Results:**

Three main themes emerged: (1) coordination of the IFI collective (2) horizontality between the collective’s members (i.e., employees and volunteers) and between the nine collaborating HR associations comprising the collective, and (3) affinity between the collective’s members and their commitment to HR. All three themes reflect one of the key features of the collective’s organization in terms of implementing HR actions, specifically the blurring of roles between partygoers, the collective’s employees and its volunteers. This role-blurring fosters the sharing of another key feature - experiential knowledge - at all levels in the collective’s organization.

**Conclusion:**

The IFI HR collective is characterized by coordination, horizontality, affinity, and the commitment of its members. Through the blurring of roles between all concerned stakeholders, experiential knowledge is welcomed and used to improve the adaptability and responsiveness of the collective’s HR actions. All these elements enable the collective to carry out relevant HR actions in party settings, despite economic and organizational challenges.

## Introduction

*InterCAARUD Festif Île-de-France* (IFI) is a collective of nine French harm reduction (HR) associations operating in the party setting in the Greater Paris region. In the scientific literature, the term “party setting” is used to describe nightlife and party contexts [1], including electronic dance music (EDM) events [2] and free techno parties, where drug use is usually normalized [3]. Various studies have analyzed festivals and rituals as intense moments of disruption and social transformation, deeply rooted in specific cultural traditions [4–7]. Drawing a parallel, in the present study, we consider that the party settings fosters shifts in normative frameworks and a multiplicity of social interactions [2, 8]. In the Greater Paris region, where the IFI operates, the party setting encompasses a wide range of legal, illegal, free and for-profit events, including festivals, parties in squats, and town fairs. Events vary in scale from smaller gatherings (a few hundred attendees) to larger ones (over 10,000 attendees).

The party setting is a specific context for people who use drugs (PWUD), as trajectories and practices tend to be more diverse than in other drug-use contexts (e.g., work-related usage, performance-related use) in terms of the substances used, routes of administration, and frequency of use [9, 10]. This is reflected in the French study OCTOPUS which highlighted several PWUD substance consumption profiles in the party setting [11]. In addition, PWUD who attend the party setting are more likely to be young, to belong to gender minorities, and to frequently experience fatigue, dehydration and other drug use complications during events. Furthermore, this PWUD sub-population tends not to use addiction services [12–14].

In the mid-1990s, free parties began to emerge throughout France. Along with the techno music movement, these events introduced a new way of partying outside of formal and authorized circuits. As “temporary autonomous zones” [15], they enabled partygoers to challenge certain constraints, particularly regarding drug use. Initially, this counterculture began on the outskirts of cities, far from traditional urban party spaces and from official health structures; accordingly, alternative care and peer-support strategies were created to provide help to partygoers in situ. Growing public health concern in the early 2000s over risk taking behaviors in the party setting (see above), motivated harm reduction (HR) advocates and free party activists to turn unofficial strategies into official HR programs specifically targeting the party setting [16]. This context was the catalyst for the recognition of experiential knowledge as a source of relevant knowledge to improve HR interventions. The notion of experiential knowledge emerged in the 1970s, with knowledge acquired through experience within self-support groups being legitimized as valid and useful [17].

It was against this backdrop that the *InterCAARUD Festif Île-de-France* (IFI) collective was created in the Paris region. Specifically, nine local associations providing HR services in various contexts^1^ joined forces in 2011 with the specific mission of providing HR services in the party setting.

Many studies evaluating HR interventions in this setting focus on drug-checking [18, 19]. Research on other types of HR interventions (e.g., distribution of HR tools, information on drugs) is less common [20, 21]. A relatively small number of evaluation studies have been conducted in specific contexts, such as alcohol consumption [22] or MDMA use [23] among partygoers. All these studies evaluated the interventions themselves; to date, no study has examined how individual and structural elements of HR organizations working in the party setting impact their interventions or how they succeed in implementing HR interventions despite economic difficulties.

After a request by the IFI, we conducted a qualitative, community-based participatory research study of the collective to investigate its organization, with a view to acquiring a better understanding of the features that enable it to provide relevant HR interventions.

Our study aimed to answer the following research questions: What features of the organization of the IFI collective enable it to provide HR interventions? Despite the specific challenges of the party setting, how do IFI members (i.e., employees and volunteers) engage in and implement relevant HR actions?

## Materials and methods

### The IFI collective

The members of the IFI collective comprise paid employees– who work as HR intervention coordinators– and volunteers. Specifically, coordinators are present on the ground at events; they supervise volunteer teams from the collective’s nine HR associations. Usually, only one association is present at any one event. This collective approach facilitates resource sharing and coordination. Coordinators also manage HR supplies and logistics.

The IFI provides several HR services; examples include materials related to drug use, sexual risks, and auditory risks; information about substances; first-aid support for minor injuries; support for partygoers who have a bad trip; a designated gender-specific physical private space to listen and to offer support to victims of sexual and gender-based violence.

At most events, the IFI will set up its own HR booth. This typically consists of a counter with information materials (e.g., brochures, posters), sometimes self-service HR supplies, a chill-out area where partygoers can rest, and the private listening and support space mentioned above. In some situations, the following services are also provided: a drug checking service in a dedicated tent, a private area for injection support in accordance with legal directives, and dedicated rest spaces for coordinators and volunteers.

### Study design

We used a community-based participatory research (CBPR) approach as it adds ethical value to the scientific process and enhances the quality of the results obtained [24]. This approach is especially useful in studies focusing on populations who are distant from health services [25], which is one of the characteristics of PWUD in the party setting [14]. The CBPR approach ensures that all stakeholders and community members who wish to participate, co-construct the research study [26]. In our study, members of the IFI collective were involved from the development of the research question to the investigation tools (e.g., interview guides), to data collection, analysis, evaluation and promotion of the results. By employing the CBPR approach, we were able to (i) obtain concrete feedback from stakeholders on the development of research tools, (ii) ensure that relevant and comprehensible language would be used in interactions with the participants, (iii) facilitate better access to participation, and (iv) observe key moments in the collective’s operations.

Members had the right to veto the use of raw data (e.g., field notes, observation reports) related to them; however, none chose to do so. Primarily, we communicated through the collective’s operational channels, as well as during the ethnographer’s on-site visits, specifically during training days and coordination meetings. A number of the collective’s members who participated in the study are co-authors of this article.

### Data collection

Data collection took place between December 2022 and December 2023 in various areas of the Greater Paris region, where the IFI collective is active. We employed an ethnographic approach. This methodological choice aligns with previous studies demonstrating the value of ethnography in public health, particularly for accessing “difficult” fields [27] where illegal or stigmatized practices, such as drug consumption [28], occur. Ethnography has also been effectively used to investigate HR strategies among queer nightlife workers [29], as well as drug use, HR, and dance club culture in Ontario [30].

### Three different data sources

Three different data sources were used: participant observation, semi-structured interviews, and focus groups; we discuss all three below.

Participant observation requires ethnographers to be physically present, to directly participate in social settings, and– in the field of drug use– to be able to overcome the specific challenges of carrying out observations (e.g., gaining trust, understanding cultural codes) [31]. When carrying out fieldwork in the EDM context, ethnographers use concepts such as “embodied participation” and “immersion” [32] to clarify their role. For the present study, the ethnographer participated directly as a volunteer in IFI’s HR actions and in training days for the collective’s volunteers; she was also present at informal moments of the collective, and observed employee coordination meetings.

Two types of ethnographic data were recorded using the pocket notebook: (i) informal exchanges and relevant events collected in real time; (ii) sketches of spatial arrangements of locations and people, facts, impressions, emotional states and feelings in real time. At the end of each day or during breaks, more detailed notes incorporating the real-time notes were written in digital format on an encrypted computer; documents were backed up on a secure server. These more detailed notes were then reviewed to write a comprehensive field journal that was subsequently used for a thematic analysis with Nvivo^®^ software (see Data Analyses below).

In line with anthropological research in general [33], and more specifically, with ethnographic studies of drug use [34], the ethnographer took photographs to complement the written field journal in order to document aspects that were difficult to capture with writing. Particular attention was given to ensuring the anonymity of individuals in photographs. A total of 124 photographs were taken.

In terms of participant observation, the ethnographer was present at the following gatherings: (i) an illegal 4-day party event (72 h of observation) and a legal 2-day event (36 h), both events numbering approximately 30,000 people; (ii) two training sessions for the collective’s volunteers (11 h and 8 h, respectively ), (iii) various volunteer meetings (10 h total), (iv) a meeting for the collective’s coordinators (8 h), and (v) informal party moments for the collective’s members (20 h total). For a more complete picture of the entire corpus of data, see Table 1.

The two other sources of data were (i) two focus groups (*n* = 17), one with IFI volunteers and one with its employees, and (ii) six in-depth individual semi-structured interviews (*n* = 6). Snowball sampling was the primary recruitment strategy for the semi-structured interviews and focus groups.

**Table 1:** Ethnographic data

Place of observation	Date	Type of fieldwork	Data	Hours
First IFI volunteer training day	03/12/22	Exploratory fieldwork; Participant observation	Ethnographic notes and informal interviews *n* = 5	12 h
Informal party moment for the collective’s members (i.e., employees and volunteers)	03/12/22	Exploratory fieldwork	Ethnographic notes and informal conversations *n* = 8; informal interviews = 4	6 h
Second IFI volunteer training day	22/04/23	Exploratory fieldwork; Participant observation	Ethnographic notes and informal conversations *n* = 6	12 h
IFI HR intervention at an illegal event	Illegal event	Participant observation	Ethnographic notes and informal conversations *n* = 18; informal interviews = 7	72 h
IFI HR intervention at a legal event	Legal Event	Participant observation	Ethnographic notes and informal interviews *n* = 9	50 h
Meeting for coordinators	03/10/23	Participant observation	Ethnographic notes and informal conversation *n* = 8	12 h
Volunteer meeting	01/11/23	Participant observation	Ethnographic notes and informal interviews *n* = 6	12 h
Community channels	01/04/23 to 01/10/23	Participant observation	Ethnographic notes	40 h
Total			*n* = 61 (*n* = 31 informal interviews)	216 h

For the interviews and focus groups, the different roles of the collective’s members were initially identified through documentary research, and later confirmed by the members themselves. By including a diverse profile of employees and volunteers, in terms of age and gender, we ensured that the study sample provided a certain degree of representativeness of the collective as a whole.

Of the 47 IFI members who participated in the collective’s HR actions in 2022, 23 participated in the focus groups and semi-structured individual interviews, while 31 participated in informal interviews. Some of the persons who took part in the semi-structured interviews also participated in informal interviews. All semi-structured interviews and focus groups were audio-recorded (with participants’ consent) and transcribed verbatim in an anonymized form.

### Data analysis

All collected data were anonymized using a random mapping table, that is to say a manually created correspondence table in which each participating IFI member was assigned a randomly generated code to ensure anonymity while preserving the link to their role (i.e., employee or volunteer) in the collective. With the support of the Nvivo^®^ 2020 software package, we performed a detailed thematic analysis of the corpus of data based on inductive coding. Drawing on grounded theory [35, 36], we selected themes using inductive logic and then prioritized until a taxonomy was obtained that could answer the research questions. We will not present all the themes here; instead, we will focus on those we consider most relevant for illustrating the specific features of the IFI collective which provide us with an understanding how it is able to implement relevant HR interventions.

### Theoretical framework

This study lies at the intersection of several major research fields: nightlife studies, the anthropology of public events, and the sociology of organizations. We briefly describe these below.

The field of nightlife studies [37, 38] explores the specific dynamics of nocturnal spaces and develops ethnographic methodologies suited to this context [39, 40], such as immersive and embodied participation [32]. In terms of the anthropology of public events, this study draws in particular on Handelman’s work [41], where nighttime events are not merely reflections of society but also play a fundamental role in structuring and transforming it. Third, to better understand how the collective’s governance shapes employees’ and volunteers’ roles, we drew on perspectives from organizational sociology [42] including volunteer sociology [43, 44]. Organizational sociology highlights that boundaries between roles within organizations is central to their functioning [45].

## Results

The age of the 23 participants in the semi-structured interviews and focus groups ranged widely from 21 to 50 years. Interestingly, volunteers tended to be younger than employees (mean age of 23 versus 30 years). With regard to gender, there were 10 men, 11 women, and two who identified as non-binary.

Three main themes emerged from our thematic analysis: (1) coordination of the IFI collective (2) horizontality between the collective’s members and between the nine collaborating HR associations comprising the collective, and (3) affinity between the collective’s members and their commitment to HR.

### Coordination of the IFI collective

According to the IFI’s founders, the main strength of the collective lies in the pooling of logistical, human, and material resources. This collective approach ensures that HR interventions can still take place even when one of the nine participating associations lacks sufficient resources for a particular event.

Employees (i.e., coordinators) have standard work contracts. If they choose to work at HR events at weekends or at nighttime, they receive time off in lieu. A coordinator from one of the collective’s nine associations can coordinate volunteer teams from other participating associations. This is one of the defining features of the IFI collective, and is rarely found in other HR organizations.

Employee coordinators and volunteers prepare HR interventions together but have different responsibilities (e.g., coordinators manage logistics, while volunteers distribute prevention messages and HR tools directly to the public), particularly in the context of interventions at legal events.

Coordinators and volunteers work together at the IFI booth. During one observed intervention at a legal event, the key role of the employee coordinating the intervention in collaboration with volunteers was particularly evident, as highlighted in the ethnographer’s field notes:

By the time I arrived, the booth was already set up with its two tents (the coordinator spent time doing that the previous day), and we only needed to unpack part of the informational material and equipment from the boxes. […] Lightning and electricity had also been arranged in advance. […] Before the official opening of the booth, we all came together to assign volunteers for the booth and for patrols [around the event], together with the coordinator. […] We spent the entire evening, right until the end of the festival (and even afterward to pack up the booth), taking turns to ensure a constant presence while also having the chance to attend some of the music event. […] In reality, I later realized that I hadn’t had any time to watch or dance at one of the many stages, and spent my breaks chatting with the other volunteers with techno music in the background. (source: field notes, 3 September 2023)

**Fig. 1:**
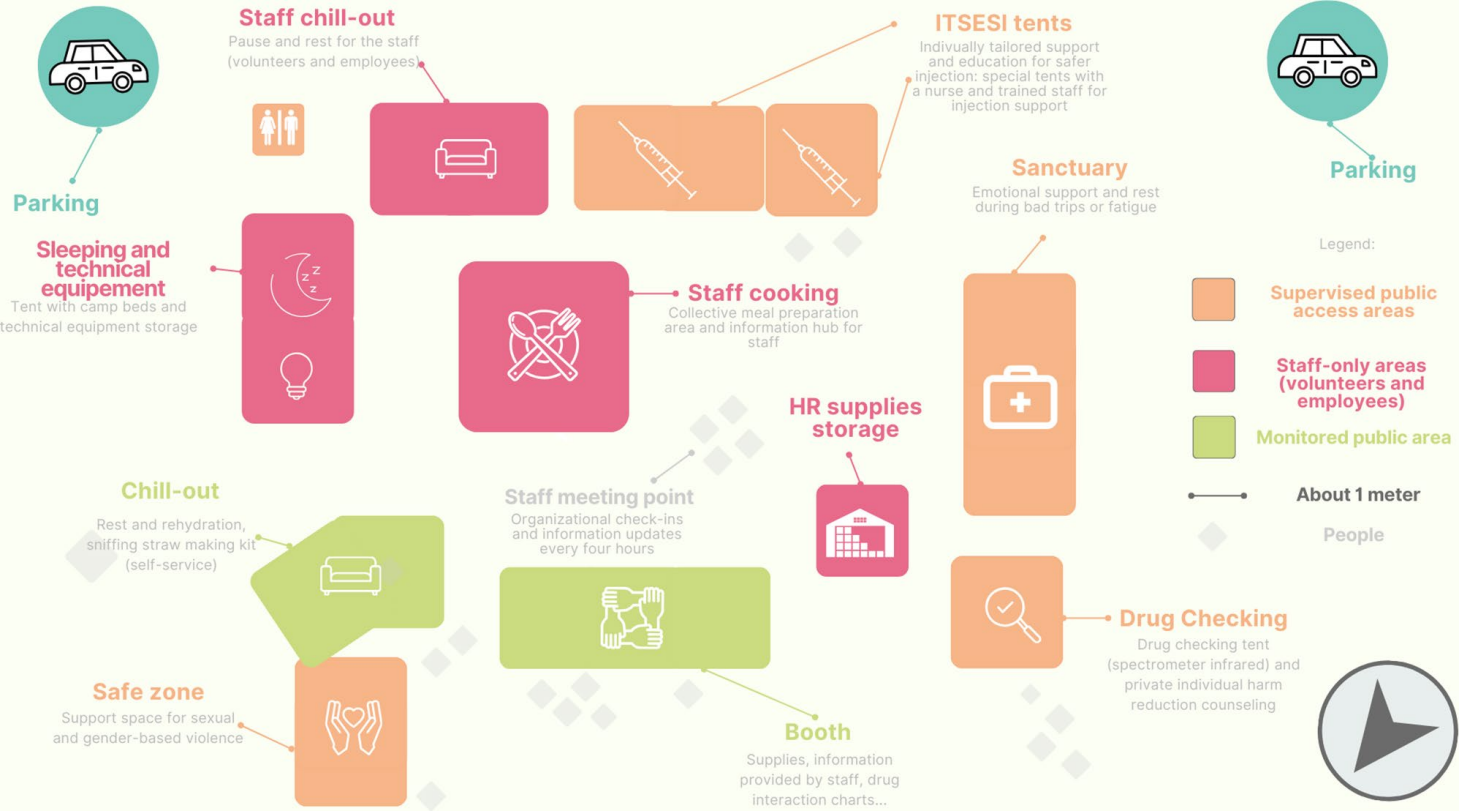
Sketch from field notes of an illegal event^2^, illustrating the layout of the IFI’s HR booth

**Fig. 2:**
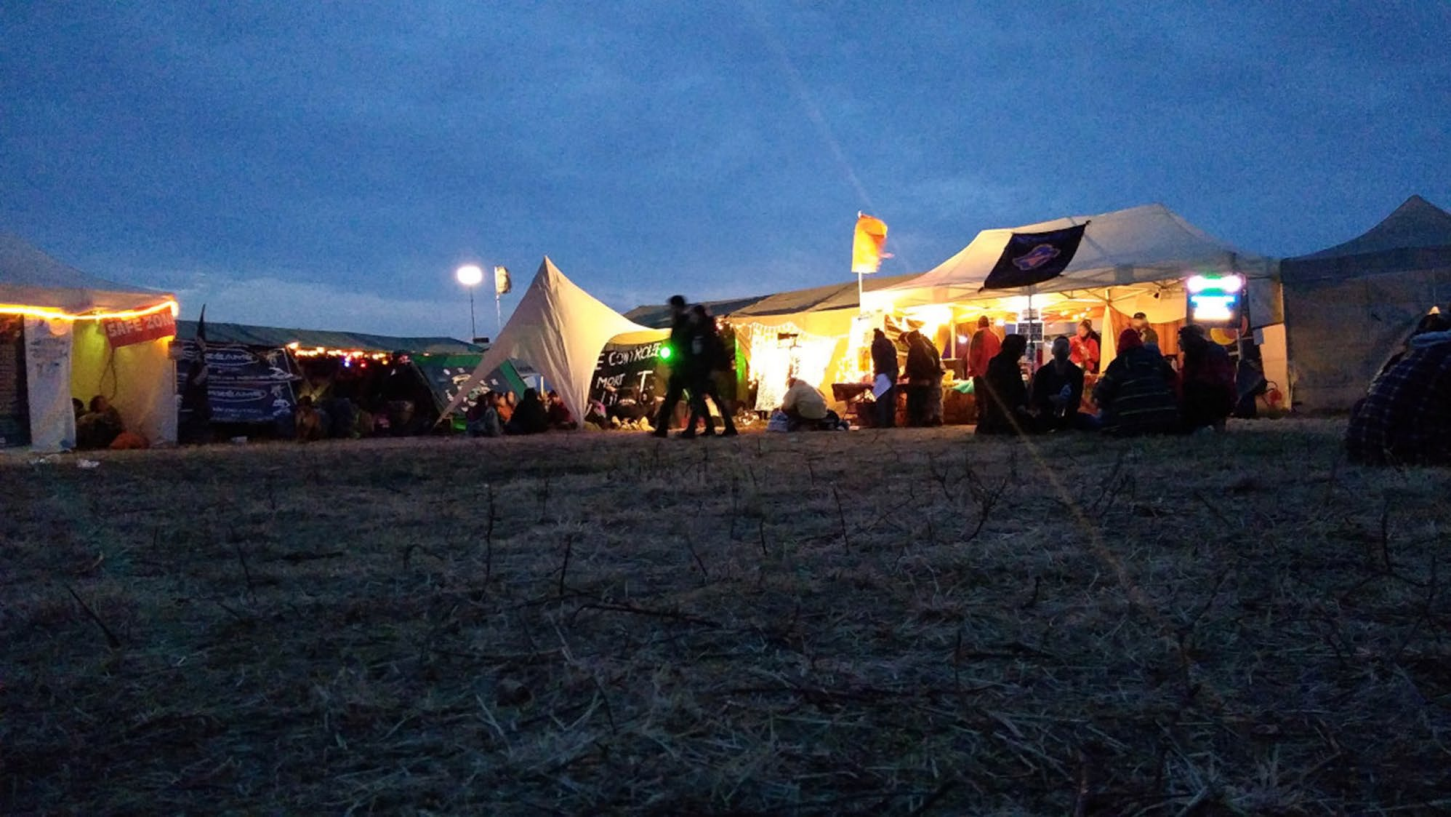
From field notes. Photography of IFI’s HR booth at an illegal free party (30,000 people), May 2023

**Fig. 3:**
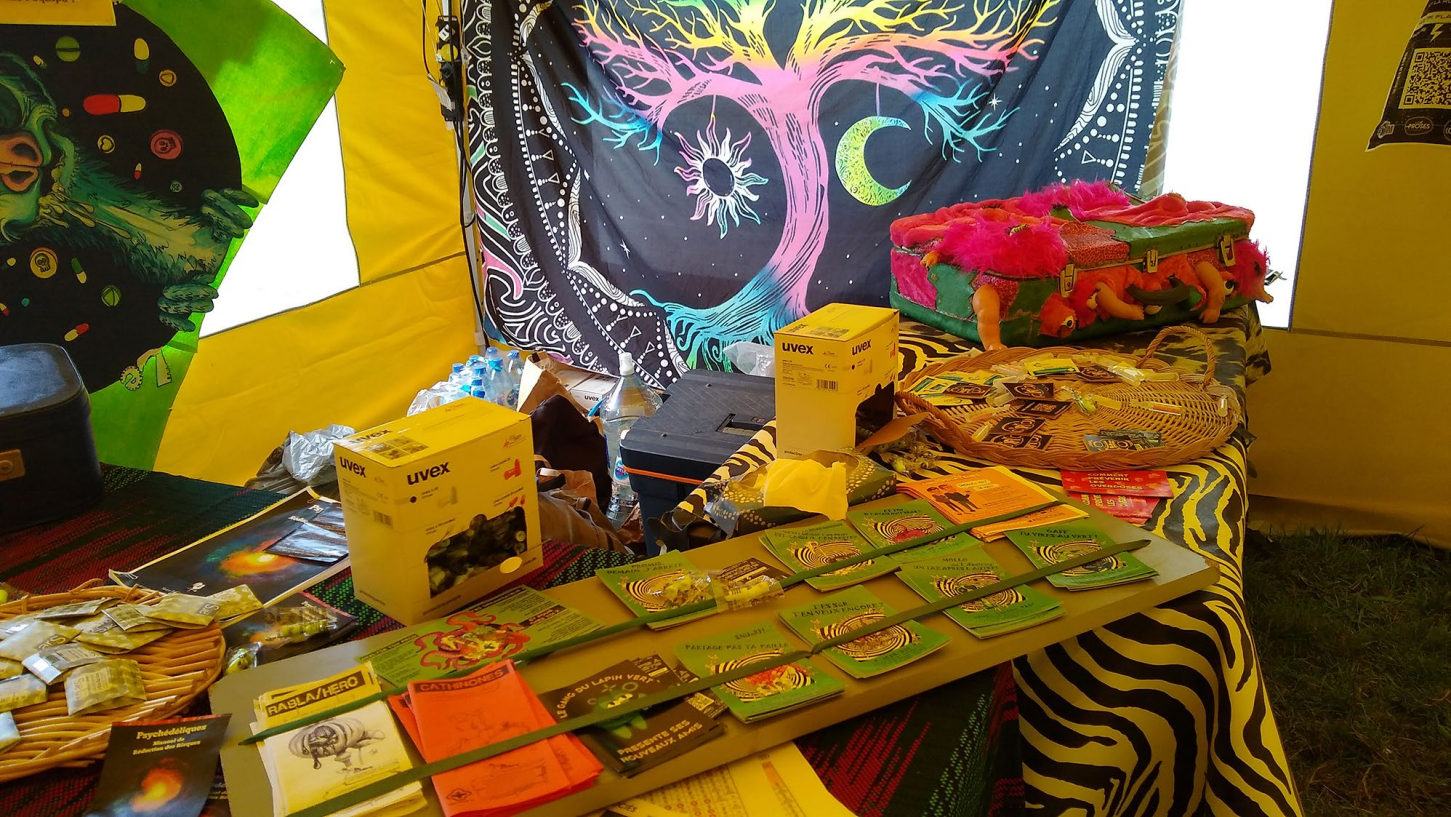
Photo from field notes: IFI booth during a legal electronic music festival (30,000 people), September 2023

The photograph in Figs. 1, 2 and 3 highlights aspects of the implementation of the IFI’s HR interventions at legal events. Generally, at these types of events, organizers are less open to the presence of organizations providing HR interventions. This is reflected in less physical space being allotted by event organizers to the IFI to set up its booth. Moreover, at legal events, some interventions are not permitted by the organisers (e.g., drug-checking, private space for consummation).

In general, persons attending legal and public events are less familiar with HR interventions. One observed legal event reflected this; many participants were unfamiliar with HR and the purpose of associations’ (i.e., in general) booths before meeting the IFI team there. Some even perceived HR as redundant and saw the presence of emergency services to be more important. Interestingly, during this same event, emergency service personnel came directly to the HR team asking for help on providing care to a person who had consumed substances the personnel were unfamiliar with. They also requested training in HR for the party setting. Similar situations were also observed at illegal events, where institutional emergency teams were initially skeptical of the IFI team’s presence but ultimately recognized the relevance of its HR approach. The ethnographer’s field notes describe the situation:

Throughout the evening, several people approached our booth asking, ‘What are you selling?’ The coordinator and other volunteers I spoke to about this clearly stated that, in general, during legal interventions — often in commercial [i.e., legal] festivals/events — organizations are very uncomfortable proposing harm reduction actions. This is partly because it would mean acknowledging the consumption of psychoactive substances, which are supposed to be prohibited on site, and secondly, they struggle to understand what harm reduction work entails (‘but we already have the Red Cross and road safety initiatives,’ the organizer of a large festival supposedly said). Moreover, the public often has difficulty locating the booth (some organizers forced us to label it “Glitter Stand” instead of “Harm Reduction Booth”), and they also struggle to understand the approach. In contrast, during illegal interventions, the members of the public I spoke to were quick to think of calling the harm reduction stand in case of problems (more so than the Red Cross security) and kept saying, ‘What you’re doing is great!’.

(source: field notes, 2 September 2023)

### Horizontality between the collective’s members and between the nine collaborating HR associations comprising the collective

The IFI collective is organized around specific roles for implementing HR in the party setting. As mentioned above, IFI employees provide a methodological framework for interventions and coordinate volunteer teams on the ground. Volunteers are not necessarily affiliated with the association (i.e., one of the nine comprising the IFI) providing HR at a specific event; they identify themselves as IFI volunteers. This approach promotes organizational flexibility and the pooling of human resources among all nine partner associations.

The following excerpt from an interview with an employee illustrates this dynamic:

the IFI, how it works: there are all these HR services which are all in the IFI. We’ve got all professionals who choose to do harm reduction at parties, and they can coordinate things [an HR action]. You only need one coordinator and then he goes with the volunteers. The volunteers can come from anywhere, from any HR structure, and can take part in any action, even if they don’t necessarily have professionals in the HR structure they’re attached to.

So each association has its own ‘pool’ of volunteers with its own meetings.

This account highlights a multi-level organization comprising both a centralized coordination role that ensures continuity in interventions, and a diversity of statuses among members. This model assures adaptability of HR actions while maintaining a structured working framework for volunteers and for employees.

In their discourses, IFI members clearly explained the various roles within the collective and how they intersect. For example, employees from one of the collective’s nine associations sometimes offered to take on the role of volunteer during an event where HR was provided by another IFI association. The following excerpt from an employee illustrates how the roles of volunteer and coordinator coexist within IFI’s actions:

Like, sometimes I go to do an HR action, and I’m not the employed coordinator; like today for example. So I don’t know what needs to be done, I don’t know the meeting times, I won’t be looking after the equipment, the booth.

Moreover, employees valued volunteers for the complex range of skills they bring to implementing HR interventions, as indicated here.:

[speaking] As an employee, for me too, volunteers are professionals.

Another employee explained that volunteers make their professional competences (particularly in medical professions) and personal skills (such as active participation in techno festival movements) available when the collective is implementing HR:

…[there are some volunteers] who study pharmacy or I don’t know, other things, and.[there are some volunteers who contribute to the IFI collective thanks to] the network of party settings they frequent.

This perception of the complex skillset which volunteers bring to interventions was shared by the volunteers themselves. In the following excerpt, the volunteer did not perceive any difference in how volunteers and employees were treated by the collective and emphasized the trust that employees placed in them:

they trust us, there’s no…. it’s horizontal actually; the coordinator is there for practical questions and all that, but they do the same work as us; they just have additional organizational responsibility but they’re the same behind the booth; I mean, it doesn’t change anything.

Our results also showed that the HR actions implemented would not be possible if there were not this dynamic of coordinator-volunteer horizontality.

Besides horizontality, working in HR in the party setting requires commitment and a genuine passion for this milieu. Some HR workers working in street-based outreach refuse to take part in nightlife interventions or to work on weekends, while IFI members find meaning in working in the party setting, as one employee explained:

some people prefer street outreach; for some, like me, it’s the party setting. For some, it’s out of the question to work at night, to work on weekends or even…. [for participate to HR party setting actions] you really have to love the party setting, love the environment, and love that crowd.

Moreover, some IFI employees had themselves been partygoers in the past, while some of the collective’s members continued to actively frequent the party setting. The latter served as a bridge between the public (and sometimes event organizers), the collective as a whole, and the collective’s employees. The importance of maintaining a connection with the world of free parties and EDM was explicitly expressed in the words of an employee who no longer frequented these settings:

Well, typically, I hardly ever go to parties anymore. If it weren’t for the volunteers, I wouldn’t know what was going on in the party setting…so, I have colleagues who still go to events, but for me it’s the volunteers; I think they’re a really important source of information. Because they’re going to keep my up to date on the events that are on, the ones which they go to; some of them are involved in organizing the music […]. So, fortunately, there are colleagues and fortunately there are volunteers.

Maintaining this connection enables the IFI to stay informed about musical events, establish contacts with organizers, and interpret the specific needs of the public for each event. More than just an adjustment to compensate for the absence of a dedicated employee for this role, this interconnected structure ensures direct, field-based insights that an external staff member, unfamiliar with the free party setting, would struggle to acquire.

Both volunteers and employees felt a close connection to partygoers and often identified with them. In our interviews, some employees working in urban HR centers explicitly expressed that they did not share the same living conditions as the highly precarious populations they provide support to. In contrast, they felt closer to the socioeconomic situation of people in the party setting (despite recognizing a certain heterogeneity across different types of events) and shared with them a passion for musical and festival culture, sometimes even a sense of community, particularly when providing HR interventions at free parties.

This sense of identification may be the result of the fact that taking on the role of volunteer did not prevent the collective’s members from also taking on the role of partygoer.

This was reflected in one volunteer’s discourse, which highlights not only the blurring of roles in the context of implementing HR actions, but also the importance of being passionate about music and EDM culture if one wants to get involved in providing HR:

Because listening to music is very important during my HR actions. It’s half volunteering, half Boom-Boom…[…] you get blasted by the sound. I think that’s very important.

This identification with partygoers - and therefore with the same cultural universe - may also come from the fact that some employees and volunteers had a history of drug use in the party setting. After extensively describing his many experiences in the party scene as a partygoer, one volunteer highlighted how these experiences contributed to his desire to get involved in HR in the party setting:

I got to know HR in the party setting because I myself went to party events and was a drug-user.

Up to this point, we have analyzed horizontality among the members of the collective; we shall now discuss horizontality among the collective’s nine different collaborating associations.

All the study participants had a very clear perception of horizontality within the collective, particularly in terms of its structural organization across the nine associations. Despite varying levels of involvement– whether in the number of interventions, employees, or volunteers– all nine were considered equal in the governance of the collective. This is clearly expressed in the discourses of both a volunteer and an employee:

In fact, there’s a horizontality between us… A horizontality in the collective.

(Volunteer)

It’s got to do with a horizontality thing, because there is room for everyone, despite the differences.

(Employee).

This horizontality reflects the initial desire in 2011 of the collaborating associations to join forces to create a collective that would implement HR in the party setting. One employee, who witnessed the creation of the collective, stressed the decision at the time to pool the different associations’ resources:

The IFI was born out of that too; that is to say that there were HR services that acted on their own, but that meant there were 2 or 3 days [a week] where the service couldn’t be staffed, and therefore, after a while, as several HR centers got to realize this a bit, they said to themselves ‘perhaps…we need to group up…’.

### Affinity between the collective’s members and their commitment to HR

The IFI members interviewed often mentioned friendship as the ‘glue’ in relationships within the collective, whether in a professional, voluntary, or partygoing (i.e., when they attended events as partygoers) context. This friendship resulted from a strong affinity among the collective’s members, as evidenced by two members with different backgrounds. One became a volunteer after meeting the HR team at student events:

I was at University478, where there were a lot of friends who were doing HR.

The other, an employee, developed strong friendships with other members who they implemented HR interventions with:

The colleagues I go to parties with [i.e., as members of the HR staff] have become friends.

In addition to affinity, commitment to HR was a recurring theme in discourses. This was evident, for example, by employees from one association (and therefore paid by that structure) helping a different association implement a HR intervention outside their working hours (i.e., taking on the role of a volunteer). HR actions in the party setting, which are often carried out under extreme conditions (high noise levels, mud, dust, or extreme summer heat), help forge strong friendships that foster collective engagement.

Working under extreme conditions or difficult hours despite their right to refuse under standard workplace safety clauses, also reflects employees’ commitment to the collective and to HR. Genuine passion for the party setting is key in this context, as highlighted by a volunteer:

At free parties it just never stops. You never really know [when you can leave]; sometimes, if you’re stuck in the convoy, you might end up going 48 hours without sleep. You have to set everything up, and you build much stronger bonds with your colleagues because you see each other all the time. You sleep in the same tents, you’re working at festivals where you don’t wash for four days, you deal with mental load, and you experience a lot together.

For them [employees from HR center], working at night or on weekends is out of the question.

Coordinating volunteer teams on the ground is a particularly intense job because of the sometimes difficult situations one has to deal with (e.g., emergencies, dealing with violence, overdoses, evacuations). IFI’s employees and volunteers develop human and professional skills to deal with these situations. The following excerpt from an employee compares party setting-based HR actions with the more routine work carried out in HR centers:

A lot of people might think it’s [i.e., working in the HR party setting] just about showing up, partying, and chatting a bit, but the most intense situations, like emergencies, dealing with violence, overdoses - even if they’re not fatal - or needing to do medical evacuation, all those things happen at these events.

Commitment to HR goes hand-in-hand with strong community bonds; IFI members choose to work in intense situations because of the profound changes that the HR interventions which they implement can make to the public’s perceptions, discourses, and practices regarding drug use. The importance of the work the collective performs, goes beyond simply distributing messages and materials. It requires personal investment, empathy, and the desire to engage with partygoers who openly share their - sometimes problematic - experiences. This is revealed in the following excerpt by a volunteer.

We don’t just hand out supplies or give harm reduction messages. It’s not just about handling people who are high or anything like that. I end up having a lot of really important conversations about people’s personal lives, personal addiction issues outside of the party setting, and stuff related to sexual violence.

For some IFI members, the motivation to get involved with HR in the party setting was related to their strong identity with the politics of techno music activists; for others, it was their passion for partying and for music. In any case, for most interviewed members, it was out of a strong commitment to and belief in HR.

## Discussion

Most of the literature on HR in the party setting focuses on the type of intervention (i.e. drug checking) and its evaluation [18, 19] or on the profiles of attendees [11]. To our knowledge, no study to date has examined the extent to which individual and structural features within an HR organization implementing interventions in the party setting influence its ability to carry out these interventions.

Three themes emerged from our analysis: coordination, horizontality, affinity & commitment. In the following paragraphs, we interpret all three through the intertwined notions of experiential knowledge and role blurring, two key features which in terms of the IFI collective’s organization, enable it to provide relevant HR interventions in the party setting.

### Coordination

In order to implement HR interventions, the collective operates through teams comprising volunteers and paid staff. However, this formal distinction between roles does not fully reflect the reality on the ground. As highlighted in our field observations and data analysis, volunteers’, employees’, and partygoers’ roles are often blurred. Rather than a fixed division of labor, IFI members shifted fluidly between functions depending on the context, their level of experience, and their personal engagement with the party setting. According to Turner’s role theory, which examines how individuals and collectives negotiate and perform overlapping social roles [46], role blurring can be understood through the lens of symbolic boundaries. In contrast, from Lamont and Molnár’s perspective, social boundaries are not rigid; rather they are continuously negotiated and redefined through interactions [47]. In the IFI collective, role blurring does not imply the absence of boundaries, but their constant reconfiguration. In order for this reconfiguration to occur, it is essential to have a framework where roles (in our case, employees, volunteers and partygoers) *can* be blurred. In the IFI, this framework is made possible by the collective’s coordination, which is based on its governance structures and the role of paid coordinators, and which ensures that individual skills are equitably recognized and redistributed.

### Horizontality

The coordination required to make role blurring possible is intrinsically linked to another core theme shaping the collective’s organization: horizontality. Although the concept of horizontality has been primarily explored in management literature in relation to organizational structures (49), it also draws meaning from activist and countercultural movements such as the 1990s techno scene [49], where it embodied values of autonomy and collective decision-making. More recently, horizontality has become a key focus in discussions on the nonprofit sector, particularly within community health associations [50]. In this perspective, Lamont and Molnár’s work [47] examined how social boundaries are negotiated and reconfigured through everyday interactions, as it is the case in the IFI collective.

### Affinity and commitment

The IFI collective also relies on affective and relational dimensions– namely, affinity and commitment– which are closely tied to the recognition of experiential forms of knowing.

In our study, friendship was the ‘glue’ in relationships within the collective. The role of friendship in various HR contexts in the party setting [51, 52], and in HR more generally [53], has been investigated in other studies. Investigating these relationships in even greater depth could provide better insight into how peer support and experiential knowledge work together to contribute to the development and Implementation of HR actions in the party setting.

In terms of IFI members’ commitment to HR, we observed that both volunteers and employees saw their involvement in the collective as a personal investment driven by passion and commitment. This was highlighted by the fact that some employees even engaged in HR outside of work hours, effectively becoming volunteers. The relationship between the collective and its volunteers is characterized by mutual benefit: the former profits from unpaid workers, while the latter profit through skill development, networking, and acquiring material advantages (e.g., free festival admissions). This reciprocal dynamic, however, also reflects a deeper tension between social mission and economic imperatives, a tension that, as Billis [54] notes, is inherent to hybrid organizations and is clearly visible in the structure of the IFI collective.

These organizational characteristics of the IFI collective facilitate the recognition and the circulation of experiential knowledge. Closely tied to role blurring, the IFI members’ experiential knowledge in the party setting is not solely based on formal employment status, but also on the possibility for this knowledge to be shared with all members of the collective.

Our study highlights that role blurring and experiential knowledge are two essential features for implementing relevant HR interventions in the party setting. However, they are not always considered important in public HR policy. We suggest greater recognition of these features if relevant and context-sensitive HR interventions are to be developed.

### Study limitations

This study has limitations. First, we did not systematically include perspectives from all the different stakeholders. For example, we did not conduct formal interviews with event organizers or with emergency response teams. Second, a larger mixed-methods approach could have provided a better understanding of the external effects of the collective (e.g., on PWUD communities, on the directors of the collective’s nine different collaborating associations, on institutional partners, on policy makers) and the impact of its HR actions on partygoers’ health and quality of life. Finally, the organizational dynamics observed within the IFI are shaped by the French legal and institutional framework, which differ from the regulatory frameworks governing HR models in other countries.

## Conclusions

This study of the organization of the IFI collective highlights two key intersecting features– role blurring and experiential knowledge– that enable it to implement relevant HR interventions tailored to public needs in the party setting. These features exist independently of the fact that to ensure the institutional continuity of the collective’s nine participating associations, the IFI must comply with the current legal HR framework. However, they are not always considered important in public HR policy.

Investigating the impact of role blurring and experiential knowledge in the institutionalization of HR in the party setting with a view to developing relevant HR interventions is an avenue for future research.

## Data Availability

No datasets were generated or analysed during the current study.

## Footnotes

1 *PROSES*, *SidaParoles 92* (through its dedicated party setting HR team called ‘The Green Bunny Gang’), *SidaParoles 78* (through its dedicated party setting HR team called ‘The High Meerkat Gang’), *MCATMS 94*, *Inter’Lud 77*, *Yucca SOS*, *Charonne Oppelia*, *Aurore 93*, and *AIDES 95*.

2 All identifying elements (first names, names of structures, places, etc.), were anonymized during transcription in order to guarantee confidentiality. For the same reason, people are cited according to their role, with a neutral gender. Essential extra information is provided in square brackets to ensure the subtleties of the discourses can be understood. Certain words have been translated differently to ensure clarity.

## Abbreviations

I.F.I.: InterCAARUD Festif Île de France
